# Acid Lipase from *Candida viswanathii*: Production, Biochemical Properties, and Potential Application

**DOI:** 10.1155/2013/435818

**Published:** 2013-11-17

**Authors:** Alex Fernando de Almeida, Sâmia Maria Tauk-Tornisielo, Eleonora Cano Carmona

**Affiliations:** ^1^Environmental Studies Center, Universidade Estadual Paulista, CEA/UNESP, Avenida 24-A, 1515 Bela Vista, 13506-900 Rio Claro, SP, Brazil; ^2^Biochemistry and Microbiology Department, Bioscience Institute, Universidade Estadual Paulista, IB/UNESP, Avenida 24-A, 1515 Bela Vista, 13506-900 Rio Claro, SP, Brazil

## Abstract

Influences of environmental variables and emulsifiers on lipase production of a *Candida viswanathii* strain were investigated. The highest lipase activity (101.1 U) was observed at 210 rpm, pH 6.0, and 27.5°C. Other fermentation parameters analyzed showed considerable rates of biomass yield (*Y*_*L*/*S*_ = 1.381 g/g), lipase yield (*Y*_*L*/*S*_ = 6.892 U/g), and biomass productivity (*P*_*X*_ = 0.282 g/h). Addition of soybean lecithin increased lipase production in 1.45-fold, presenting lipase yield (*Y*_*L*/*S*_) of 10.061 U/g. Crude lipase presented optimal activity at acid pH of 3.5, suggesting a new lipolytic enzyme for this genus and yeast in general. In addition, crude lipase presented high stability in acid conditions and temperature between 40 and 45°C, after 24 h of incubation in these temperatures. Lipase remained active in the presence of organic solvents maintaining above 80% activity in DMSO, methanol, acetonitrile, ethanol, acetone, 1-propanol, isopropanol, and 2-propanol. Effectiveness for the hydrolysis of a wide range of natural triglycerides suggests that this new acid lipase has high potential application in the oleochemical and food industries for hydrolysis and/or modification of triacylglycerols to improve the nutritional properties.

## 1. Introduction

Lipases (triacylglycerol acyl hydrolase, EC 3.1.1.3) are responsible for the hydrolysis of triglyceride ester bonds into diglycerides, monoglycerides, fatty acids, and glycerol using a complex phenomenon of interfacial activation. Lipases present a number of unique characteristics, including substrate specificity, stereospecificity, and the ability of the resolution of racemic mixtures and synthesis of esters bonds in nonaqueous media [1]. The potential for industrial applications of lipases comprises the industry of additives (modification of aromas), food (monoacylglycerols and diacylglycerols), fine chemistry (ester synthesis), detergents (hydrolysis of fats), wastewater treatment (decomposition and removal of oleaginous substances), leather (fat removal from animal skin), pharmaceutical, and medical area (medicines, digestive aid, and enzymes for diagnosis) [2–5].

Lipases are ubiquitous and indispensable for triacylglycerols bioconversion in nature. They occur in plants, animals, and microorganisms [6], but the most lipases commercially produced are isolated from fungi, yeast, and bacteria [5, 7, 8]. Microbial enzymes are often more utilized than enzymes from plants or animals because of the diversity of catalytic activities, the high yields, ease of genetic manipulation, regular supply due to absence of seasonal variations, and rapid growth of microorganisms on inexpensive media [9]. Microbial lipases are biocatalysts that have interesting characteristics such as action under mild conditions, stability in organic solvents, high substrate specificity, and regio- and enantioselectivity [3, 10].

The use of the submerged culture is advantageous because of the facility of sterilization and process control in these systems. Depending on the strain and the culture conditions, the enzyme can be constitutive or inducible, showing different production. Submerged fermentation processes for the lipolytic enzymes production usually employ renewable low-cost hydrophobic substrates such as vegetable oil, poultry fat, tallow, waste fats, soap stocks, rapeseed oil, and grease containing waste water [11]. The use of these carbon sources for synthesis of high-added value products, as single-cell protein, microbial lipids, organic acids, biosurfactants, and lipases, is considered of great economic and ecological significance [12].

Environmental factors such as oxygen, agitation, temperature, and pH are important parameters that affect extracellular lipase production and must be considered in submerged process. Temperature and pH can be determined in shake flasks fermentations [13, 14], but agitation and oxygenation of the culture should be specifically studied in the course of the scale-up process [15–17]. Another important point considered in lipases studies is the biochemical characterization of the crude enzymes. This is frequently used to know the catalytic properties of enzymes, since in general no large quantities of pure enzymes can be obtained after purification without these data [18]. Moreover, in some lipase industrial applications this is not necessary because homogenous preparations are not required [19].

In a previous study, a strain of *Candida viswanathii *was capable to produce lipase and biomass efficiently when cultured in a large diversity of natural triacylglycerols [20]. In this work, the influence of environmental factors such as agitation, pH, and temperature, and surfactants supplementation were investigated to improve the lipase production by this yeast. In addition, the lipase produced under the best culture conditions was biochemically characterized and the potential application for triacylglycerols hydrolysis was evaluated.

## 2. Materials and Methods

### 2.1. Microorganisms and Growth Conditions

*C. viswanathii* strain is available in the Culture Collection of the Environmental Studies Center, CEA/UNESP, Brazil. *C. viswanathii* was cultivated on malt extract agar (MEA) for 3 days, at 28°C, for inoculum preparation. Liquid medium was prepared using Vogel's medium, with 1.5% (w/v) olive oil and 0.2% (w/v) yeast extract as single carbon and nitrogen sources, respectively, according to the conditions established previously [20]. Erlenmeyer flasks (125 mL) containing 25 mL of medium were inoculated with 1.0 mL of cells suspension (1.0 × 10^7^ cells/mL) and incubated at different conditions as indicated subsequently. All cultures were developed in triplicate and the results were assayed for biomass, maximum lipase activity, biomass yield on substrate, lipase yield on substrate, biomass productivity, and specific rate of lipase production.

Biomasses were separated from the fermentation broth by filtration. Cell-free broth was used for lipase activity determination. Biomasses were dried at 105°C until constant mass.

### 2.2. Effect of Agitation on Lipase Production

The culture was carried out in an orbital shake at 150 rpm, 180 rpm, and 210 rpm. Initial pH was adjusted to 6.0 and the incubation temperature was 27.5°C. Fermentation parameters were monitored in different intervals for 96 h.

### 2.3. Effect of Initial pH and Temperature on Lipase Production

The effect of initial pH on lipase production was analyzed from 3.0 to 10.0. The initial medium pH was adjusted by the addition of NaOH 1 M or HCl 1 M. Cultures were carried out for 72 h, 210 rpm orbital shake, at 27.5°C.

The temperature influence on lipase production was varied from 20 to 40°C, with intervals of 2.5°C. Cultures were carried out for 72 h, 210 rpm orbital shake, and pH 6.0.

### 2.4. Effects of Emulsifiers and Surfactants on Lipase Production

Different surfactants (Tween 20, Tween 80, and Triton X-100) and emulsifiers (gum Arabic, soybean lecithin, and sodium deoxycholate) on lipase production were added to the medium at 0.1% (w/v). Cultures were carried out for 72 h, 210 rpm orbital shake, and pH 6.0 at 27.5°C.

## 3. Analytical Methods

### 3.1. Lipase Activity Assay

Lipase activity was assayed with *ρ*-nitrophenyl-palmitate (*ρ*NPP) as substrate [21]. *ρ*NPP was first dissolved in 0.5 mL of dimethyl sulfoxide and then diluted to 50 mM with 50 mM sodium phosphate buffer pH 7.0 containing 0.5% Triton X-100. The hydrolysis of *ρ*NPP was determined discontinuously at 37°C by releasing *ρ*-nitrophenol (*ρ*NP). After 5 min of preincubation of 0.9 mL of this substrate solution in water bath, the reaction was started by addition of 0.1 mL of appropriately diluted sample. The reaction was stopped at different intervals (1 and 2 min) by heat shock (90°C, 1 min), followed by addition of 1 mL of saturated sodium tetraborate solution. The absorbance was measured at 405 nm and the activity was determined according to the standard curve carried out with *ρ*-nitrophenol (*ρ*NP molar extinction coefficient: 1.8 × 10^4^ M^−1 ^cm^−1^). Controls were prepared without enzyme. One unit of enzyme activity was defined as the amount of enzyme that releases 1 *μ*mol of *ρ*NP per mL per min.

### 3.2. Protein Analysis

The protein was determined by the Lowry method [22], using bovine serum albumin as standard.

## 4. Enzyme Characterization

### 4.1. Optimum pH and pH Lipase Stability

Enzyme activity was measured at 37°C in different pH values using glycine-HCl 0.05 M buffer from 2.0 to 3.0 and McIlvaine buffer from 3.0 to 8.0. Enzyme stability was carried out with the same buffers, except from 8.6 to 10.0 when glycine-NaOH was used. Enzyme preparation was diluted in each buffer (1 : 2, v/v) and incubated for 24 h at 10°C. 

### 4.2. Optimum Temperature and Lipase Thermal Stability

The optimum temperature was determined by incubation of the reaction mixture from 25 to 60°C, in the McIlvaine buffer pH 4.0. For thermal stability, the enzyme was incubated for different periods of time at 40, 45, 50 and 60°C in McIlvaine buffer pH 4.0 and the residual activity was determined in McIlvaine buffer pH 3.5 at 40°C.

### 4.3. Effect of Solvents on Lipase Activity

Methanol, ethanol, acetone, isopropanol, acetonitrile, 1-propanol, 2-propanol, and dimethylsulfoxide (DMSO) were added at 1% (v/v) and 10% (v/v) to the reaction medium. The relative activities were expressed as a percentage of the control. 

### 4.4. Effect of Substances on Lipase Stability

Crude enzyme was incubated at 40°C for 1 h in the presence of substances ZnSO_4_, MgSO_4_, NaCl, BaCl_2_, CoCl_2_, NH_4_Cl, Pb(CH_3_COO)_2_, CaCl_2_, and EDTA at final concentration of 10 mM and 20 mM. 

### 4.5. Hydrolysis of Triacylglycerols

Hydrolysis of pure (tributyrin, triolein) or natural oils and fats (canola, castor, lard, linseed, maize, palm, poultry, olive, soybean, and sunflower) was developed at 40°C by following the titration of fatty acids released. The oils (10%, w/v) were emulsified in McIlvaine buffer pH 4.0, containing 5% (w/v) Triton X-100. The reaction was started by adding 1 mL of sample to 5 mL of this emulsion and then maintained for 30 min at 300 rpm orbital agitation. The reaction was interrupted by adding 16 mL of an acetone : ethanol solution (1 : 1, v/v) to the mixture. The fatty acids released were titrated to pH 11 with a 0.05 M NaOH solution. One unit of enzyme activity was defined as the amount of enzyme that releases 1 *μ*mol of fatty acid per mL per min. The results were expressed as percentage of triolein hydrolyzed.

## 5. Results and Discussion

### 5.1. Effect of Agitation Speed on Lipase Production

**Figure 1:**
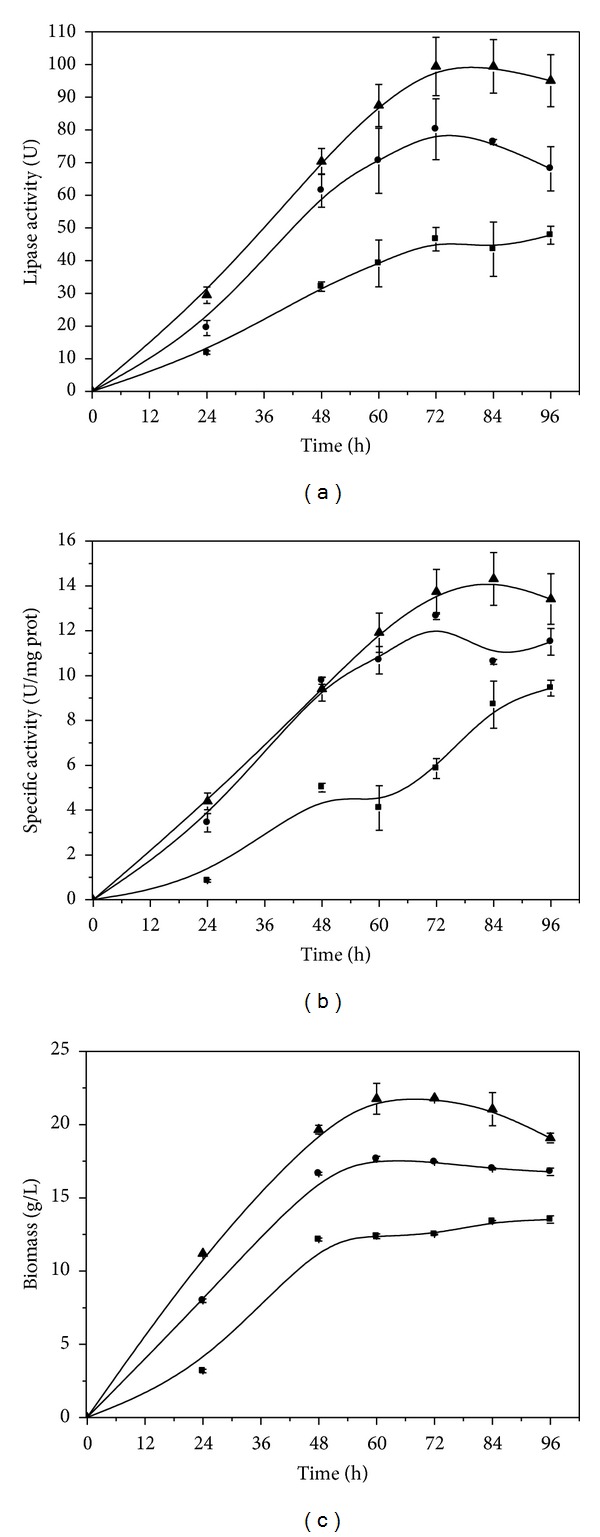
Effect of the agitation speeds in orbital shake on lipase production (a), specific activity (b), and* Candida viswanathii* growth (c). Culture conditions: liquid cultures were carried out in Vogel's medium with 1.5% (w/v) olive oil and 0.2% (w/v) yeast extract, at pH 6.0 and 28°C. (■) 150 rpm; (*⚫*) 180 rpm; (▲) 210 rpm.

Preliminary studies demonstrated very low growth rate and no lipase production until 72 h (3 days) of culture in stationary cultures. The subsequent experiments evaluated the agitation effect on the *C. viswanathii* growth and lipase production, and specific activity was determined. Three agitation speeds were assayed, and lipase production was found to be growth-associated with maximum after 72 h cultivation (Figures 1(a) and 1(c)). The peaks of lipase production and biomass were verified at 210 rpm with values of 99.4 U and 21.8 g/L, respectively. For cultures grown at 150 rpm and 180 rpm, the lipase production corresponded to 46.50 U and 80.00 U and the cell growth to 12.1 g/L and 17.4 g/L, respectively. The highest specific activity was also observed at 210 rpm but after 84 h of cultivation (Figure 1(b)). The decrease in the extracellular lipase activity observed after 72 h cultivation can be attributed to the hydrolytic action of proteases [23], to the decrease in cell growth, or to adsorption of the enzyme produced at the aqueous-organic interface [24].

Other fermentation parameters analyzed in cultures performed at 210 rpm for 72 h showed biomass yield (*Y*_*X*/*S*_) 1.456 g/g, lipase yield (*Y*_*L*/*S*_) 6.638 U/g, biomass productivity (*P*_*X*_) 0.303 g/h, and specific rate of lipase production (*q*_*L*_) 0.062 U of lipase/g of biomass·h (Table 1). The biomass yield obtained was higher than those found in the literature, which present values considered satisfactory for single-cell protein fermentation from fatty acids, around 0.5–0.7 g/g [12, 25, 26].

The results obtained in shake flasks are similar to those obtained using agitation speeds in bioreactors [17, 24, 27]. Brozzoli et al. [28] showed that lipase production by *Candida cylindracea *NRRL Y-17506 in olive mill wastewater was significantly affected by stirring speed using aeration of 1.0 vvm. Elibol and Ozer [15] reported that the variation in agitation speed for the *Rhizopus arrhizus *cultures resulted in a change in oxygen transfer rate, which in turn affected the rate and extent of cell growth and lipase production in shake flasks. However, in this study, the agitation speeds did not reduce period for production and increase lipase yield. According to Takaç et al. [24] cultures performed in batch bioreactor show considerable advantages over shake flasks cultivation in terms of lipase production and yield. Gulati et al. [29] reduced the time period for lipase production by *Aspergillus terreus* from 96 h in shake flask to 54 h in a bioreactor with control of dissolved oxygen and agitation. Nevertheless, shake flask culture is an important step for cell growth and lipase production comprehension and can play an important role in the scale-up process.

### 5.2. Effect of pH and Temperature on Lipase Production

The initial pH of the fermentation medium is an important physical parameter that affects microbial growth and enzymes synthesis by *Candida *spp. strains [28, 30]. Cultivation in different initial pH values was used to evaluate the lipase production by *C. viswanathii *(Table 2). Changes in the pH were observed during growth in shake flasks due to the consumption of the medium nutrients or products formation. The lipase production increased with increasing pH up to 6.0, in which the lipase production was higher (100.0 U). For cultures in neutral and alkaline conditions, the lipase production decreased, whereas *C. viswanathii *biomass levels remained almost constant (around 15 g/L). An optimal initial pH for lipase production by the yeast *Pichia lynferdii* was found at 7.0 [31] and for the bacteria *Bacillus pumilus *was 9.0 [32].

The highest specific activity (13.8 U/mg protein) and biomass (18.9 g/L) were also verified in these culture conditions. Fermentation parameters analyzed showed high biomass and lipase yields in pH 6.0 (*Y*_*X*/*S*_ = 1.312 g/g and *Y*_*L*/*S*_ = 6.807 U/g, resp.) as well as specific rate lipase production (*q*_*L*_ = 0.079 U of lipase/g of biomass·h). Ali et al. [30] reported maximum lipase production by *C. lipolytica* in this same pH. An initial pH of 4.5 was reported as optimum for lipase production by continuous fermentation of *Yarrowia lipolytica* [33]. 

**Figure 2:**
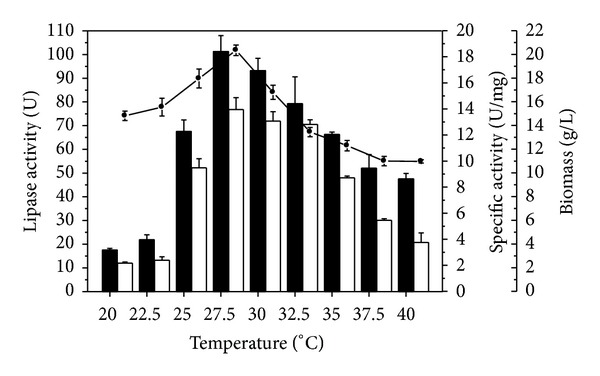
Effect of temperature on growth and lipase production by *C. viswanathii*. Culture conditions: cultures were carried out in Vogel's medium with 1.5% (w/v) olive oil and 0.2% (w/v) yeast extract and agitated at 210 rpm, at pH 6.0. (■) lipase activity, (□) specific activity, and (*⚫*) biomass.

The cultivation temperature ranging from 20 to 40°C and its effects in the cell growth and lipase production were also analyzed, maintaining the conditions previously established (72 h cultures, 210 rpm, and pH 6.0). When the incubation temperature was increased, lipase production, specific activity, and cell growth also increased up to 27.5°C (Figure 2). The microbial growth at 27.5°C corresponded to 20.3 g/L, decreasing substantially up to 40°C (10.9 g/L). In this temperature, the values of lipase production and specific activity were 101.2 U and 13.9 U/mg, respectively. Both lipase production and specific activity remained at high levels until 32.5°C, decreasing significantly after this temperature. The highest biomass and lipase yields as well as maximal biomass (*P*_*X*_ = 0.282 g/h), were also observed at 27.5°C (*Y*_*X*/*S*_ = 1.381 g/g and *Y*_*L*/*S*_ = 6.892 U/g), (Table 3). However, the highest specific rate of lipase production was at 32.5°C (*q*_*L*_ = 0.079 U of lipase/g of biomass·h) although operational cost is inferior at temperature next to room temperature.

These results are consistent with those observed by Lin et al. [16] which demonstrated that temperature control during submerged cultures is a critical factor that can greatly reduce the productivity of the system even with relatively small variations. *C. viswanathii *was sensitive to variations of 2.5°C for enzyme production and growth. In other studies, several microorganisms were investigated to produce lipase at different temperatures [26, 34] and most of them presented optimum temperature for enzyme production between 25 and 30°C [30, 35, 36].

### 5.3. Effect of Surfactants and Emulsifiers on Lipase Production

Surfactant or emulsifier addition during the fermentation process has often been shown to enhance extracellular lipase production due to changes in the permeability of the cell or surfactant effects on cell-bound lipase. Nevertheless, surfactants and emulsifiers do not always increase lipase production, and their effects appear to depend on both surfactant and the strain studied [36]. The influence of surfactants Tween 20, Tween 80, and Triton X-100 and emulsifiers gum Arabic, soybean lecithin, and sodium deoxycholate on cell growth and lipase production is presented in Table 4. Soybean lecithin was the only one that increased lipase production (147.5 U). The parameters, lipase yield (*Y*_*L*/*S*_ = 10.061 U/g) and specific rate of lipase production (*q*_*L*_ = 0.114 U of lipase/g of biomass·h), were considerably superior to the results observed without emulsifier (control). The parameters biomass yield (*Y*_*X*/*S*_ = 1.226 g/g) and biomass productivity (*P*_*X*_ = 0.250 g/h) decreased with the addition of this emulsifier. Soybean lecithin is composed of specific phospholipids, triglycerides and other nonphospholipids nontoxic compounds [37], which may have been metabolized by organisms. Other surfactants or emulsifiers used in the culture media show a deleterious effect on lipase production. Triton X-100 greatly reduced lipase and biomass production (5.75 U and 5.6 g/L, resp.). Domínguez et al. [36] reported that surfactants did not significantly increase lipase production by *Y. lipolytica*, but Pogori et al. [38] reported that *Rhizopus chinensis* lipase production decreased when surfactants were added to the culture medium.

## 6. Biochemical Characterization

### 6.1. Effect of pH on Activity and Stability

**Figure 3:**
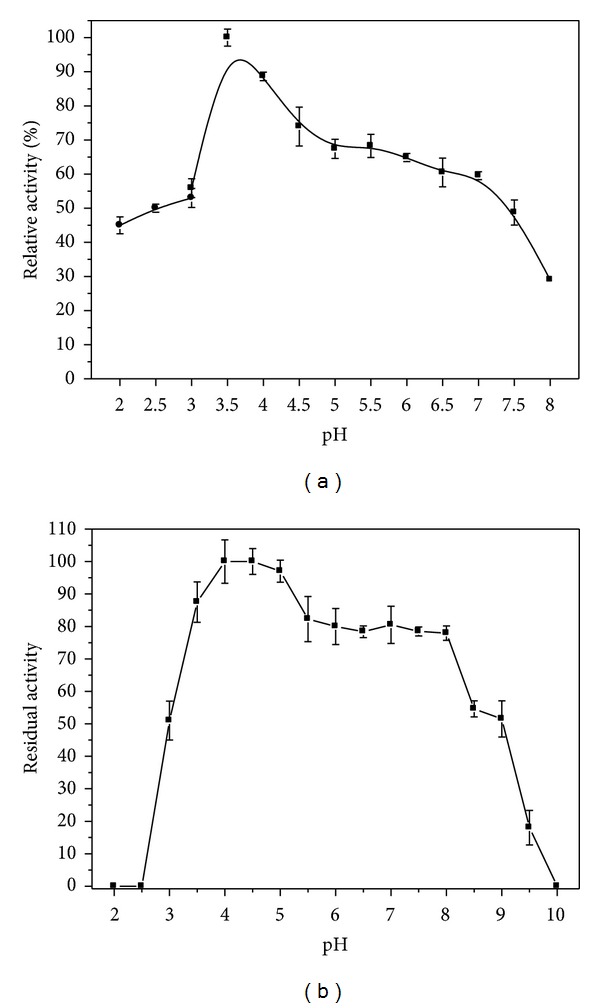
Optimal pH (a) and pH stability (b) of the crude *C. viswanathii *lipase. Assay conditions: 0.05 M glycine-HCl buffer from 2.0 to 3.0, McIlvaine buffer from 3.0 to 8.0, and 0.05 M glycine-NaOH from 8.0 to 10. Lipase activity assays were carried out at 37°C (a) and in McIlvaine buffer pH 3.5, at 37°C (b).

The effects of pH on lipase activity and stability are shown in Figure 3. The lipase activity (100%) was maximal at pH 3.5 (Figure 3(a)). The activity decreased significantly up to pH 5.0 and slightly from this pH up to 7.0, reducing markedly from 7.0 to 8.0. At pH range 2.0-3.0, lipase activity shows 45 to 53%, respectively. The enzyme was more stable in the pH range from 4.0 to 5.0, retaining almost 100% of activity after 24 h. At pH range from 5.5 to 8.0 the residual activity remained around 80% (Figure 3(b)). Residual activity remained above 50% at pH 3.0 and at 8.5–9.0; however, at pH range from 2.0 to 2.5 and 10.0 the enzyme was completely inactivated.

The optimum acid pH of *C. viswanathii *lipase activity is different from other *Candida *lipases that presented optimal pH range in alkaline conditions. *Candida rugosa* lipase had optimal activity at pH 7.5 [39]. Lipase from *Candida antarctica *showed the highest activity at pH 8.0 and the enzyme was stable at pH range of 7.0–9.0 after 24 h [40]. *Candida cylindracea *lipases A and B showed similar pH profiles using tributyrin as substrate, with optimum activity around pH 7.0. However, the decrease in activity at pH 8.0 was more pronounced in lipase B, whereas lipase A was less active at pH 5.0 [41]. An acid lipase with optimal activity at pH 2.5 was produced by *Aspergillus niger* NCIM 1207 [42]. The lipolytic activity of this strain decreased significantly when the pH was increased to 4.0 and its stability at alkaline pH range of 8.0–11.0 retained 100% of its original activity after incubation for 24 h. Maximal activity of *Pseudomonas gessardii *lipase produced with beef tallow was observed at pH 5.0; however above pH 7.0, the activity rapidly decreased, retaining 33% of its activity at pH 9.0 [43]. This enzyme was also produced using slaughterhouse waste showing maximum pH activity at 3.5 and stability between pH 2.5 and pH 5.5 [44].

### 6.2. Effect of Temperature on Activity and Thermal Stability

**Figure 4:**
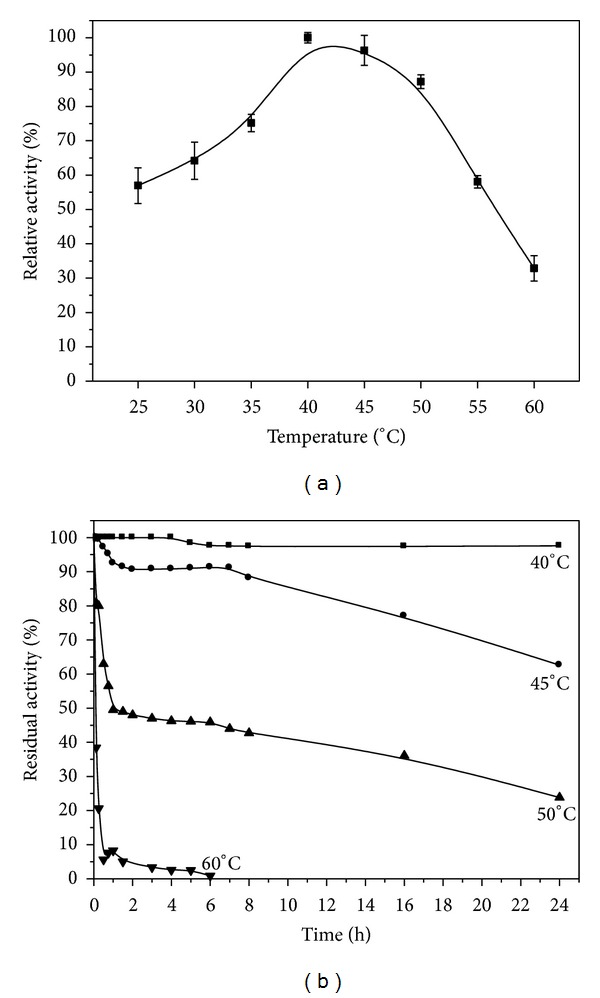
Optimal temperature (a) and thermal stability (b) of the crude *C. viswanathii *lipase. Assay conditions: McIlvaine buffer pH 3.5 (a). The enzymatic preparation was incubated at (■) 40, (*⚫*) 45, and (▲) 50°C, without substrate. The residual lipase activity was assayed with McIlvaine buffer, pH 3.5, at 40°C (b).

*C. viswanathii* lipase activity increased with temperature from 25°C up to 40°C (Figure 4(a)). The highest activity was observed at 40°C (100%), and elevated activity was also found at 45°C (96.3%) and 50°C (87.2%). Lipase activity rapidly reduced to 32.8% at 60°C and retained 56.9% of its activity at temperature of 25°C. Most fungal lipases present maximal activity at temperatures ranging from 30 to 60°C. *C. antarctica *lipase showed optimum activity at 35°C [40]. Mateos Diaz et al. [45] showed that the maximal activity of the lipase produced by *Rhizopus homothallicus *in submerged fermentation or solid state fermentation differed between 30 and 40°C. *Fusarium oxysporum *produced an alkaline lipase with maximum activity at 50°C [46]. The highest lipolytic activity against *ρ*NPP of the *Penicillium aurantiogriseum *was observed at 60°C [47]. 

The crude *C. viswanathii *lipase retained 99.8% of its activity after 24 h at 40°C (Figure 4(b)), showing 66.9% of its activity after 72 h (data not shown). At 45°C, 62.6% of lipase activity was recovered after 24 h, while, at 50°C, 50% of residual activity was observed after 0.9 h. The enzyme was not stable at temperature of 60°C, and only 8.3% residual activity was verified after 1 h of incubation. Purified *C. antarctica *lipase showed optimal activity at 35°C and maximal stability at 30°C. This enzyme showed a rapid decrease in activity at 45°C, after 1 h of incubation [40]. Ramani et al. [44] suggested that the thermal stability at comparatively higher temperature may be due to the influence of factors such as broad pH stability, metal ions, and the rigidity in the lipase structure. 

### 6.3. Effects of Organic Solvents on Lipase Activity

Lipase stability in organic solvents is an essential prerequisite for lipase applications in organic synthesis [48], since synthetic reactions with enzymes are often performed in organic solvents to shift the thermodynamic equilibrium toward synthesis [49]. *C. viswanathii *lipase activity was determined after addition of polar organic solvents in the reaction at final concentration of 1% (v/v) and 10% (v/v) (Table 5). At 1% (v/v) concentration, methanol, ethanol, 1-propanol, and DMSO had no effect on lipase activity. In the presence of acetonitrile, acetone, isopropanol, and 2-propanol at 1%, *C. viswanathii* lipase retained approximately 90% of its activity. At 10% (v/v), methanol and DMSO weakly affected the lipase activity and ethanol had a moderate effect under this condition. However, isopropanol, acetone, 2-propanol, 1-propanol, and acetonitrile drastically reduced the lipase activity. Ogino and Ishikawa [50] reported that direct contact of a polar organic solvent in monophasic systems with an enzyme can result in severe enzyme structure distortion, rapid denaturation, and even complete inactivation.

### 6.4. Effect of Substances and Surfactants on Lipase Stability

The effect of different metal ions and surfactants on the activity of the lipase is shown in Table 6. Among the metal ions assayed, only NaCl increased the activity at 10 mM and 20 mM (110.4% and 117.4%, resp.), whereas MgSO_4_, BaCl_2_, NH_4_Cl, and CaCl_2_ had no significant effect on the activity. Usually, fungal lipases can be activated by adding Mg^2+^, Ca^2+^, NH_4_, and Ba^2+^ ions to the reaction media [45, 51]. However, the crude *C. viswanathii *lipase was not activated by these ions. ZnSO_4_ and CoCl_2_ inhibited the activity of both 10 and 20 mM. ZnSO_4_ was previously reported as an inhibitor of lipase from *Penicillium *sp. [14], whereas CoCl_2_ inhibited the lipase from *Rhizopus homothallicus* [45]. The addition of the chelating agent EDTA did not affect enzyme activity, indicating that *C. viswanathii* lipase is not a metalloenzyme as reported for other lipases such as those from *Penicillium *sp. [14] and *Aspergillus awamori *[52].

Lipase activity was not affected by gum Arabic. In the presence of Tween 80, the highest inhibition was observed at 5% (60.5%). Tween 20 at 1% and 5% inhibited the activity to 88.2% and 52.5%, respectively. SDS completely inactivated the enzyme and Triton X-100 was a strong lipase inhibitor, reducing the activity to 30.3% and 26.9% at both concentrations. The lipase from *A. awamori* was also inhibited by nonionic detergents [52]. The inhibition of the lipase by an ionic surfactant, such as SDS, can be due the formation of complexes with protein in solution altering the conformational stability and the hydrophobicity of the protein surface. Additionally, partial or complete unfolding of the tertiary protein structure can be affected, due to additional hydrophobic interaction [53].

### 6.5. Hydrolytic Activity on Triacylglycerols

**Figure 5:**
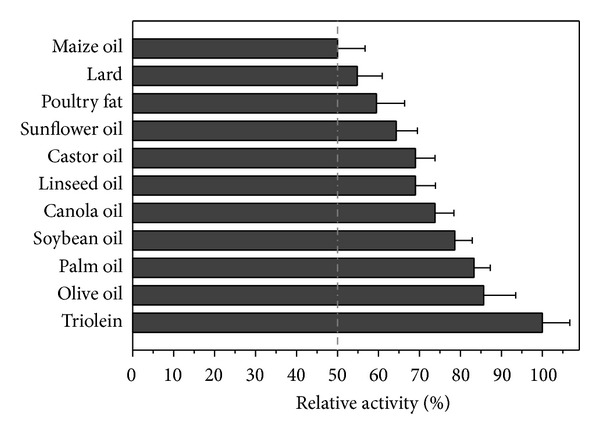
Hydrolysis of triacylglycerols by crude *Candida viswanathii* lipase. Assay conditions: triacylglycerols hydrolysis was carried out in McIlvaine buffer pH 4.0 with 5% (w/v) Triton X-100 and hydrolysis activities were assayed by titration method.

The hydrolytic activity of the lipase on pure and natural triacylglycerols is shown in Figure 5. The highest hydrolytic activity was observed for triolein (100%), olive oil (85.7%), palm oil (83.3%), soybean oil (78.6%), canola oil, castor oil (69.0%), and sunflower oil (64.3%). Intermediary hydrolytic activity was observed for poultry fat (59.5%), lard (54.8%), and maize oil (50.0%). Crude *C. viswanathii *lipase showed the lowest activity on tributyrin (45.5%). These results suggest that this enzyme is more active on triacylglycerol with long chain fatty acids being capable of hydrolyzing a broad spectrum of esters of fatty acid chain lengths. Similar results were found for lipases from *Penicillium camembertii *[54], *Colletotrichum gloeosporioides *[55], and* Pseudomonas gessardii *[43].

## 7. Concluding Remarks

The present study demonstrated the importance of controlling physical parameters for lipase production by *C. viswanathii*. Agitation speed, temperature, and initial pH are essential to produce 101 U/L of crude lipase. The lipase production was even more increased by supplementation with soybean lecithin. The biochemical characterization of the crude *C. viswanathii* lipase revealed that this enzyme shows different properties when compared to those from other *Candida *spp. Optimal activity at acid pH of 3.5 suggests a new lipolytic enzyme for this genus and for yeast in general. In addition, crude lipase presented high stability in acid conditions and was highly stable at 40 and 45°C, remaining active in the presence of organic solvents as DMSO and methanol. The enzyme was not activated by metal ions and the activity was preserved in the presence of gum Arabic for 1 h at 40°C. *C*. *viswanathii* lipase presented a broad specificity for triacylglycerols hydrolysis suggesting that this enzyme can be applied in lipid digestion and biotransformation.

## Figures and Tables

**Table 1:** Fermentation parameters of the *C. viswanathii *cultures performed for 72 h.

Speed rotation (rpm)	Biomass (g/L)	Lipase (U)	*Y*_*X*/*S*_ (g/g)	*P*_*X*_ (g/h)	*Y*_*L*/*S*_ (U/g)	*q*_*L*_ (U/g·h)
150	12.5	46.6	0.834	0.173	3.108	0.051
180	17.4	80.2	1.164	0.242	5.350	0.064
210	21.8	99.4	1.456	0.303	6.638	0.062

*Y*_*X*/*S*_: biomass yield on substrate consumed; *P*_*X*_: biomass productivity; *Y*_*L*/*S*_: lipase yield on substrate consumed; *q*_*L*_: specific rate of lipase production.

**Table 2:** Effect of culture pH on growth and lipase production by *C. viswanathii*.

Initial pH	Final pH	Biomass (g/L)	Specific activity (U/mg prot)	Lipase activity (U)	*Y*_*X*/*S*_ (g/g)	*P*_*X*_ (g/h)	*Y*_*L*/*S*_ (U/g)	*q*_*L*_ (U/g·h)
3.0	5.8 ± 0.0	11.4 ± 0.1	5.10 ± 0.78	40.5 ± 4.2	1.024	0.158	3.639	0.049
4.0	6.5 ± 0.0	16.4 ± 0.2	6.86 ± 0.63	58.0 ± 4.5	1.310	0.227	4.496	0.049
5.0	6.6 ± 0.0	16.2 ± 0.3	8.90 ± 0.88	79.5 ± 5.0	1.205	0.225	5.915	0.068
6.0	6.6 ± 0.1	18.9 ± 0.1	13.82 ± 0.44	100.0 ± 3.0	1.312	0.262	6.807	0.073
7.0	7.0 ± 0.1	14.8 ± 0.9	3.83 ± 0.38	33.0 ± 2.2	1.329	0.205	2.320	0.031
8.0	7.6 ± 0.0	15.4 ± 0.1	2.33 ± 0.22	21.2 ± 0.7	1.127	0.214	1.618	0.019
9.0	7.9 ± 0.1	15.0 ± 0.4	2.47 ± 0.37	19.7 ± 2.7	1.469	0.208	1.934	0.018
10.0	8.1 ± 0.0	14.7 ± 0.5	2.47 ± 0.26	22.0 ± 1.7	1.577	0.204	2.364	0.020

Culture conditions: Vogel's liquid medium with 1.5% (w/v) olive oil and 0.2% (w/v) yeast extract at 28°C, agitated at 210 rpm, for 72 h. *Y*_*X*/*S*_: biomass yield on substrate consumed; *Y*_*L*/*S*_: lipase yield on substrate consumed; *P*_*X*_: biomass productivity; *q*_*L*_: specific rate of lipase production.

**Table 3:** Fermentations parameters of the *C. viswanathii* cultures in different temperatures.

Temperature (°C)	*Y*_*X*/*S*_ (g/g)	*P*_*X*_ (g/h)	*Y*_*L*/*S*_ (U/g)	*q*_*L*_ (U/g·h)
20.0	1.230	0.193	1.435	0.016
22.5	1.151	0.219	1.798	0.022
25.0	1.263	0.250	4.814	0.052
27.5	1.381	0.282	6.892	0.070
30.0	1.348	0.243	6.475	0.077
32.5	1.014	0.193	5.839	0.079
35.0	0.882	0.173	4.853	0.070
37.5	0.967	0.152	4.435	0.066
40.0	1.023	0.148	4.667	0.062

*Y*_*X*/*S*_: biomass yield on substrate consumed; *Y*_*L*/*S*_: lipase yield on substrate consumed; *P*_*X*_: biomass productivity; *q*_*L*_: specific rate of lipase production.

**Table 4:** Effect of the surfactants and emulsifiers on growth and lipase production by *C. viswanathii*.

Emulsifiers (0.1%, w/v)	Lipase activity (U)	Specific activity (U/mg prot)	Biomass (g/L)	*Y*_*X*/*S*_ (g/g)	*P*_*X*_ (g/h)	*Y*_*L*/*S*_ (U/g)	*q*_*L*_ (U/g·h)
Control (no surfactant or emulsifier)	100.00 ± 3.00	13.82 ± 0.44	18.90 ± 0.92	1.456	0.303	6.638	0.062
Tween 20	77.50 ± 7.75	11.31 ± 1.14	17.81 ± 0.91	1.231	0.247	5.360	0.060
Tween 80	32.50 ± 2.75	5.07 ± 0.45	16.60 ± 0.85	1.164	0.230	2.280	0.027
Triton X-100	5.75 ± 0.50	0.89 ± 0.08	5.55 ± 0.62	0.776	0.077	0.804	0.014
Gum Arabic	65.00 ± 5.25	1.53 ± 0.16	16.83 ± 0.30	1.408	0.233	5.439	0.055
Soybean lecithin	147.50 ± 4.25	2.33 ± 0.74	17.97 ± 0.36	1.226	0.250	10.061	0.114
Sodium deoxycholate	52.50 ± 4.25	0.52 ± 0.04	14.11 ± 1.04	1.033	0.196	3.849	0.051

Culture conditions: Vogel's liquid medium with 1.5% (w/v) olive oil and 0.2% (w/v) yeast extract at 28°C, agitated at 210 rpm, pH 6.0, for 72 h. *Y*_*X*/*S*_: biomass yield on substrate consumed; *Y*_*L*/*S*_: lipase yield on substrate consumed; *P*_*X*_: biomass productivity; *q*_*L*_: specific rate of lipase production.

**Table 5:** Effect of organic solvents on lipase activity produced by *C. viswanathii*.

Organic solvents	Log *P* values	Relative activity (%)	
		1%	10%
Control (no organic solvent added)		100 ± 4.5	100 ± 5.6
DMSO	−1.378	98.9 ± 6.9	91.8 ± 6.2
Methanol	−0.764	102.6 ± 5.9	94.0 ± 5.3
Acetonitrile	−0.394	89.8 ± 7.1	16.8 ± 5.2
Ethanol	−0.235	101.9 ± 6.2	60.0 ± 7.5
Acetone	−0.208	88.0 ± 5.4	31.8 ± 4.9
1-Propanol	0.250	109.1 ± 9.1	18.6 ± 9.7
Isopropanol	0.074	91.1 ± 6.3	32.4 ± 5.4
2-Propanol	0.070	88.1 ± 6.5	29.2 ± 9.5

Assay conditions: lipase activities were assayed on *p*NPP hydrolysis using McIlvaine buffer pH 3.5, at 40°C. Log *P*: logarithm of the partition coefficient (*P*) in octanol/water two-phase system indicates the solvents hydrophobicity. DMSO: dimethylsulfoxide.

**Table 6:** Effect of substances on *Candida viswanathii* lipase stability.

	Relative activity (%)	
Substances	10 mM	20 mM

Control (no substance added)	100 ± 4.8	100 ± 4.8
ZnSO_4_	84.4 ± 3.6	60.0 ± 10.0
MgSO_4_	99.9 ± 3.9	96.5 ± 4.0
BaCl_2_	106.7 ± 3.6	98.6 ± 3.7
CoCl_2_	60.8 ± 7.4	42.7 ± 1.0
NaCl	110.4 ± 5.9	117.4 ± 6.8
NH_4_Cl	106.8 ± 4.2	104.4 ± 3.74
Pb(CH_3_COO)_2_	95.1 ± 5.0	94.2 ± 6.3
CaCl_2_	103.2 ± 4.4	96.6 ± 4.9
EDTA	103.7 ± 4.2	98.6 ± 6.1

Emulsifiers	1%	5%

SDS	ND	ND
Tween 20	88.2 ± 9.5	52.5 ± 4.9
Tween 80	93.8 ± 8.6	60.5 ± 5.8
Triton X-100	30.3 ± 6.7	26.9 ± 8.7
Gum Arabic	107.3 ± 4.8	102.0 ± 5.1

Assay conditions: lipase activities were assayed on *p*NPP hydrolysis after 1 h of incubation using McIlvaine buffer pH 3.5, at 40°C, without substrate.

## References

[1] Houde A, Kademi A, Leblanc D (2004). Lipases and their industrial applications: an overview. *Applied Biochemistry and Biotechnology Part A*.

[2] Feltes MMC, de Oliveira D, Block JM, Ninow JL (2013). The production, benefits, and applications of monoacylglycerols and diacylglycerols of nutritional interest. *Food and Bioprocess Technology*.

[3] Rigo E, Ninow JL, Tsai SM (2012). Preliminary characterization of novel extra-cellular lipase from *Penicillium crustosum* under solid-state fermentation and its potential application for triglycerides hydrolysis. *Food and Bioprocess Technology*.

[4] Fernandez-Lafuente R (2010). Lipase from Thermomyces lanuginosus: uses and prospects as an industrial biocatalyst. *Journal of Molecular Catalysis B*.

[5] Li N, Zong M-H (2010). Lipases from the genus *Penicillium*: production, purification, characterization and applications. *Journal of Molecular Catalysis B*.

[6] Sharma R, Chisti Y, Banerjee UC (2001). Production, purification, characterization, and applications of lipases. *Biotechnology Advances*.

[7] Salihu A, Alam MZ, AbdulKarim MI, Salleh HM (2012). Lipase production: an insight in the utilization of renewable agricultural residues. *Resources, Conservation and Recycling*.

[8] Contesini FJ, Lopes DB, MacEdo GA, Nascimento MDG, Carvalho PDO (2010). *Aspergillus* sp. lipase: potential biocatalyst for industrial use. *Journal of Molecular Catalysis B*.

[9] Hasan F, Shah AA, Hameed A (2009). Methods for detection and characterization of lipases: a comprehensive review. *Biotechnology Advances*.

[10] Treichel H, de Oliveira D, Mazutti MA, Di Luccio M, Oliveira JV (2010). A review on microbial lipases production. *Food and Bioprocess Technology*.

[11] Fickers P, Nicaud JM, Gaillardin C, Destain J, Thonart P (2004). Carbon and nitrogen sources modulate lipase production in the yeast Yarrowia lipolytica. *Journal of Applied Microbiology*.

[12] Papanikolaou S, Chevalot I, Galiotou-Panayotou M, Komaitis M, Marc I, Aggelis G (2007). Industrial derivative of tallow: a promising renewable substrate for microbial lipid, single-cell protein and lipase production by *Yarrowia lipolytica*. *Electronic Journal of Biotechnology*.

[13] Dutta S, Ray L (2009). Production and characterization of an alkaline thermostable crude lipase from an isolated strain of *Bacillus cereus* C7. *Applied Biochemistry and Biotechnology*.

[14] Dheeman DS, Antony-Babu S, Frías JM, Henehan GTM (2011). Purification and characterization of an extracellular lipase from a novel strain Penicillium sp. DS-39 (DSM 23773). *Journal of Molecular Catalysis B*.

[15] Elibol M, Ozer D (2000). Influence of oxygen transfer on lipase production by Rhizopus arrhizus. *Process Biochemistry*.

[16] Lin E-S, Wang C-C, Sung S-C (2006). Cultivating conditions influence lipase production by the edible Basidiomycete *Antrodia cinnamomea* in submerged culture. *Enzyme and Microbial Technology*.

[17] Salihu A, Alam MZ, Abdulkarim MI, Salleh HM (2011). Effect of process parameters on lipase production by *Candida cylindracea* in stirred tank bioreactor using renewable palm oil mill effluent based medium. *Journal of Molecular Catalysis B*.

[18] De La Casa RM, Sinisterra JV, Sánchez-Montero JM (2006). Characterization and catalytic properties of a new crude lipase from *C. rugosa*. *Enzyme and Microbial Technology*.

[19] Gupta P, Upadhyay LSB, Shrivastava R (2011). Lipase catalyzed-transesterification of vegetable oils by lipolytic bacteria. *Research Journal of Microbiology*.

[20] Almeida AF, Taulk-Tornisielo SM, Carmona EC (2012). Influence of carbon and nitrogen sources on lipase production by a newly isolated *Candida viswanathii* strain. *Annals of Biotechnology*.

[21] Yang J, Koga Y, Nakano H, Yamane T (2002). Modifying the chain-length selectivity of the lipase from *Burkholderia cepacia* KWI-56 through in vitro combinatorial mutagenesis in the substrate-binding site. *Protein Engineering*.

[22] Lowry OH, Rosebrough NJ, Farr AL, Randal RJ (1951). Protein measurement with the Folin phenol reagent. *The Journal of Biological Chemistry*.

[23] Puthli MS, Rathod VK, Pandit AB (2006). Optimization of lipase production in a triple impeller bioreactor. *Biochemical Engineering Journal*.

[24] Takaç S, Ünlü AE, Erdem B (2010). Oxygen transfer strategy modulates the productions of lipase and esterase enzymes by Candida rugosa. *Journal of Molecular Catalysis B*.

[25] Kamzolova SV, Morgunov IG, Aurich A (2005). Lipase secretion and citric acid production in Yarrowia lipolytica yeast grown on animal and vegetable fat. *Food Technology and Biotechnology*.

[26] Darvishi F, Nahvi I, Zarkesh-Esfahani H, Momenbeik F (2009). Effect of plant oils upon lipase and citric acid production in *Yarrowia* lipolytica yeast. *Journal of Biomedicine and Biotechnology*.

[27] Dominguez A, Pastrana L, Longo MA, Rúa ML, Sanroman MA (2005). Lipolytic enzyme production by *Thermus thermophilus* HB27 in a stirred tank bioreactor. *Biochemical Engineering Journal*.

[28] Brozzoli V, Crognale S, Sampedro I, Federici F, D’Annibale A, Petruccioli M (2009). Assessment of olive-mill wastewater as a growth medium for lipase production by *Candida cylindracea* in bench-top reactor. *Bioresource Technology*.

[29] Gulati R, Saxena RK, Gupta R (2000). Fermentation and downstream processing of lipase from *Aspergillus terreus*. *Process Biochemistry*.

[30] Ali S, Rafi H, Ikram-Ul-Haq I-U (2010). Production of an extracellular lipase from *Candida lipolytica* and parameter significance analysis by Plackett-Burman design. *Engineering in Life Sciences*.

[31] Kim H-R, Kim IN-H, Hou CT, Kwon K-IL, Shin B-S (2010). Production of a novel cold-active lipase from *Pichia lynferdii* Y-7723. *Journal of Agricultural and Food Chemistry*.

[32] Sangeetha R, Geetha A, Arulpandi I (2011). Pongamia pinnata seed cake: a promising and inexpensive substrate for production of protease and lipase from *Bacillus pumilus* SG2 on solid-state fermentation. *Indian Journal of Biochemistry and Biophysics*.

[33] Deive FJ, Sanromán MA, Longo MA (2010). A comprehensive study of lipase production by *Yarrowia lipolytica* CECT 1240 (ATCC 18942): from shake flask to continuous bioreactor. *Journal of Chemical Technology and Biotechnology*.

[34] Yu H, Han J, Li N, Qie X, Jia Y-M (2009). Fermentation performance and characterization of cold-adapted lipase produced with *Pseudomonas* Lip35. *Agricultural Sciences in China*.

[35] Kumar R, Mahajan S, Kumar A, Singh D (2011). Identification of variables and value optimization for optimum lipase production by *Bacillus pumilus* RK31 using statistical methodology. *New Biotechnology*.

[36] Domínguez A, Deive FJ, Sanromán MA, Longo MA (2003). Effect of lipids and surfactants on extracellular lipase production by *Yarrowia lipolytica*. *Journal of Chemical Technology and Biotechnology*.

[37] Scholfield CR (1981). Composition of soybean lecithin. *Journal of the American Oil Chemists Society*.

[38] Pogori N, Cheikhyoussef A, Xu Y, Wang D (2008). Production and biochemical characterization of an extracellular lipase from *Rhizopus chinensis* CCTCC M201021. *Biotechnology*.

[39] Khor HT, Tan NH, Chua CL (1986). Lipase-catalyzed hydrolysis of palm oil. *Journal of the American Oil Chemists’ Society*.

[40] Adamczak M (2003). Synthesis, properties, and application of lipase from *Candida antartica* for high yield monoacylglycerol biosynthesis. *Polish Journal of Food Nutriton Science*.

[41] Rúa ML, Díaz-Murino T, Fernández VM, Otero C, Ballesteros A (1993). Purification and characterization of two distinct lipases from *Candida cylindracea*. *Biochimica et Biophysica Acta*.

[42] Mhetras NC, Bastawde KB, Gokhale DV (2009). Purification and characterization of acidic lipase from *Aspergillus niger* NCIM 1207. *Bioresource Technology*.

[43] Ramani K, Kennedy LJ, Ramakrishnan M, Sekaran G (2010). Purification, characterization and application of acidic lipase from *Pseudomonas gessardii* using beef tallow as a substrate for fats and oil hydrolysis. *Process Biochemistry*.

[44] Ramani K, Chockalingam E, Sekaran G (2010). Production of a novel extracellular acidic lipase from *Pseudomonas gessardii* using slaughterhouse waste as a substrate. *Journal of Industrial Microbiology and Biotechnology*.

[45] Mateos Diaz JC, Rodríguez JA, Roussos S (2006). Lipase from the thermotolerant fungus *Rhizopus homothallicus* is more thermostable when produced using solid state fermentation than liquid fermentation procedures. *Enzyme and Microbial Technology*.

[46] Dos Prazeres JN, Cruz JAB, Pastore GM (2006). Characterization of alkaline lipase from *Fusarium oxysporum* and the effect of different surfactants and detergents on the enzyme activity. *Brazilian Journal of Microbiology*.

[47] Lima VMG, Krieger N, Mitchell DA, Fontana JD (2004). Activity and stability of a crude lipase from *Penicillium aurantiogriseum* in aqueous media and organic solvents. *Biochemical Engineering Journal*.

[48] Doukyu N, Ogino H (2010). Organic solvent-tolerant enzymes. *Biochemical Engineering Journal*.

[49] Dheeman DS, Frias JM, Henehan GTM (2010). Influence of cultivation conditions on the production of a thermostable extracellular lipase from *Amycolatopsis mediterranei* DSM 43304. *Journal of Industrial Microbiology and Biotechnology*.

[50] Ogino H, Ishikawa H (2001). Enzymes which are stable in the presence of organic solvents. *Journal of Bioscience and Bioengineering*.

[51] Supakdamrongkul P, Bhumiratana A, Wiwat C (2010). Characterization of an extracellular lipase from the biocontrol fungus, *Nomuraea rileyi* MJ, and its toxicity toward *Spodoptera litura*. *Journal of Invertebrate Pathology*.

[52] Xia J-L, Huang B, Nie Z-Y, Wang W (2011). Production and characterization of alkaline extracellular lipase from newly isolated strain *Aspergillus awamori* HB-03. *Journal of Central South University of Technology*.

[53] Delorme V, Dhouib R, Canaan S, Fotiadu F, Carrière F, Cavalier J-F (2011). Effects of surfactants on lipase structure, activity, and inhibition. *Pharmaceutical Research*.

[54] Tan T, Zhang M, Xu J, Zhang J (2004). Optimization of culture conditions and properties of lipase from *Penicillium camembertii* Thom PG-3. *Process Biochemistry*.

[55] Colen G, Junqueira RG, Moraes-Santos T (2006). Isolation and screening of alkaline lipase-producing fungi from Brazilian savanna soil. *World Journal of Microbiology and Biotechnology*.

